# Evidence of clinical efficacy of a first generation CD19 CAR T cell in B cell malignancies

**DOI:** 10.1002/jha2.731

**Published:** 2023-06-24

**Authors:** Kyrillus S. Shohdy, Manon Pillai, Ryan Guest, Dominic Rothwell, Natalia Kirillova, Shien Chow, David Gilham, Fiona Thistlethwaite, Robert Hawkins

**Affiliations:** ^1^ Division of Cancer Sciences The University of Manchester and The Christie NHS Foundation Trust Manchester UK; ^2^ Department of Clinical Oncology Cairo University Cairo Egypt; ^3^ Cancer Research UK Manchester Institute Cancer Biomarker Centre The University of Manchester Manchester UK; ^4^ Clinical and Experimental Immunotherapy Group Institute of Cancer Sciences, Manchester Academic Healthcare Science Centre Manchester UK; ^5^ Cellular Therapeutics Limited Manchester UK

**Keywords:** B cell malignancies, CAR‐T cell therapy, first‐generation, Phase I trial

## Abstract

The persistence and reactivity of CAR T cells were enhanced by adding co‐stimulatory domains, which is the basis of currently approved CAR‐T cell therapies. However, this comes at the expense of increasing toxicities from the strong cytokine release effect. This is the first report from anti‐CD19 CAR‐T cell therapy with a single activation domain to show a favourable safety profile and clinical efficacy with two patients who achieved durable responses up to 28 months in a cohort with heavily pretreated B cell malignancies.

## 1 INTRODUCTION

Preclinical studies implied that second‐generation CAR T cells bearing activatory and co‐stimulatory signaling domains were superior to first‐generation CAR T cells that bore a single T cell activation domain alone [1]. Whilst the science underlying this observation remains valid with the current licensed CAR T based on the second‐generation design [2, 3, 4], first‐generation CAR T were not extensively tested in combination with pre‐conditioning chemotherapy, which may have contributed to their perceived lack of clinical efficacy. We report here the analysis of the phase 1 clinical trial documenting, we believe, the first observation of clinical efficacy of a first generation CD19 CAR T combined with pre‐conditioning chemotherapy and supplemented with IL‐2 in patients with B cell malignancies.

## 2 PATIENTS AND METHODS

The study included patients who had histologically confirmed CD19‐positive non‐Hodgkin lymphoma with evidence of persistent or recurrent disease after previous treatment. The in vitro and in vivo evidence of the activity of our engineered CAR T cells is provided in the Supplementary Information File. Regarding the CAR construct, the scFv was isolated from the murine hybridoma HD37 and cloned in fusion with the CD3ζ receptor [5], and inserted into the pMP71 vector. A truncated (t)CD34 gene was co‐expressed using the 2A cleavage system to provide a cell surface marker to facilitate the identification of the transduced cells. Hereafter we will refer to the engineered CAR T cells as aCD19z T cells.

**FIGURE 1:**
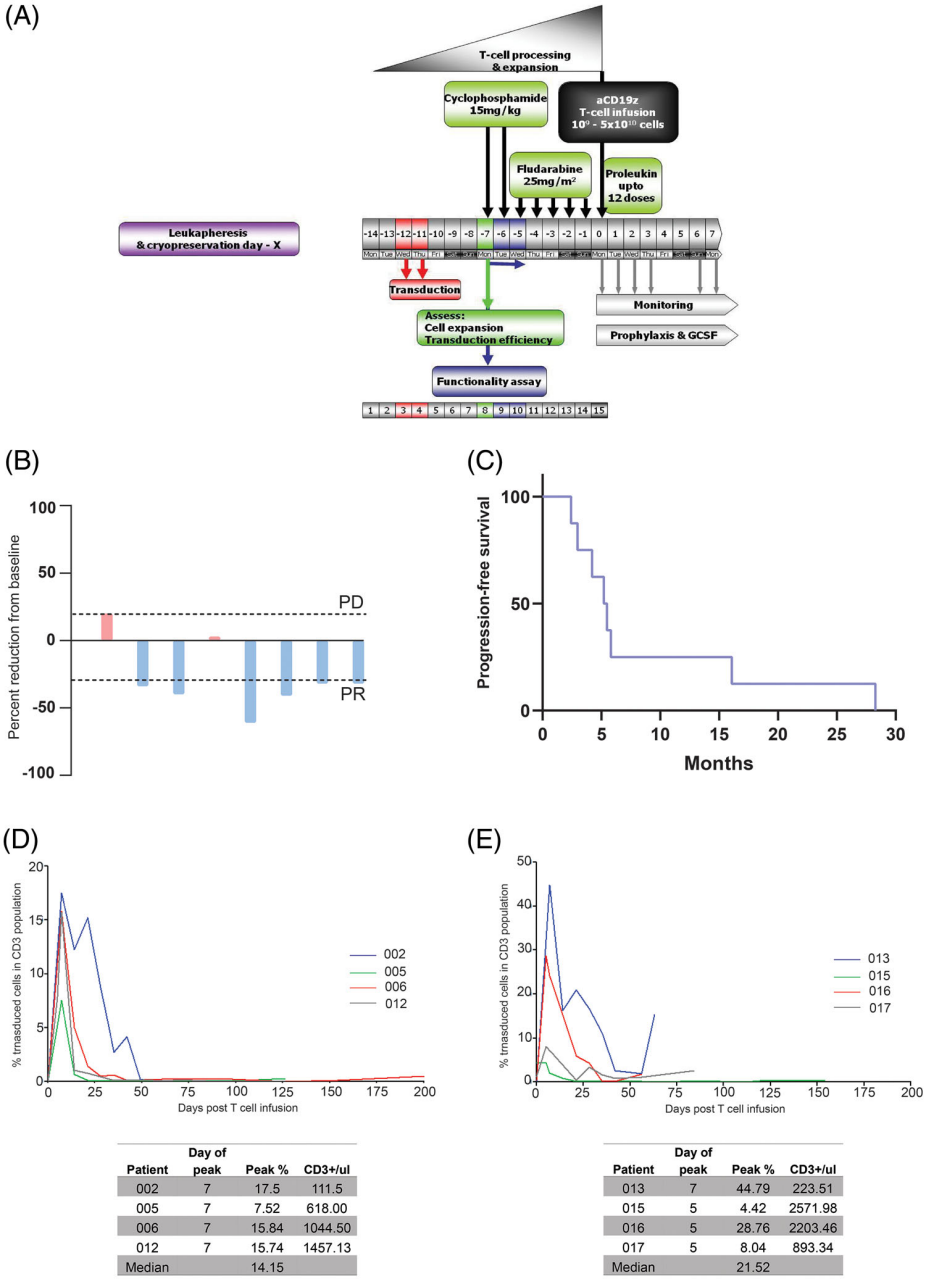
(A) flowchart showing the design, timing and procedures involved in the trial, including leukapheresis, product manufacturing, hospital admission, lymphdepleting chemotherapy, CAR T cells and IL2 administration, (B) Waterfall plot showing the percentage of tumour reduction from baseline in the eight patients, the blue color indicates partial response. (C) Kaplan–Meier curve showing the progression‐free survival of the included patients. (D) Plot showing the percent of transduced cells in CD3 population in peripheral blood after CAR T cell infusion in cohort 1. (E) Percent of transduced cells in cohort 2. The peak percentage for each patient is indicated in the boxes below the plots.

Patients received pre‐conditioning chemotherapy followed by the aCD19z T cells and then intravenous IL2. All patients were followed up till death to assess the safety and tolerability of this approach. Pre‐conditioning chemotherapy included fludarabine 25 mg/m2 from day −5 to day −1 and cyclophosphamide 15 mg/kg from day −7 to −6. Patients received aCD19z T cells on day 0. IL2 was given at a dose of 100,000 IU/kg every 8 h for up to 12 doses (Figure 1a). Safety was monitored according to the National Cancer Institute's Terminology Criteria for Adverse Events version 4.03 (NCI‐CTCAE v.4.03) and evaluated by a Data Safety Monitoring Board to assess subsequent cell dose escalation. These assays were performed prior to infusion of the final aCD19z T‐cell product, including T‐cell count and viability (CD3+ve, Annexin‐V‐ve 7AAD‐ve), detectable tCD34 expression (>20% of the CD3+ve cells must express *tCD34) and CD25 expression (Transduced cells (CD19z+) should upregulate CD25 expression on Raji cells 2‐fold more than non‐transduced cells (CD19z‐) (CD25+ and 7‐AAD‐ve cells) (Supplementary Table S1).

## 3 RESULTS AND CONCLUSIONS

Seventeen patients were screened, with eight patients eligible and having their cells successfully manufactured. The eight patients had completed treatment with an aCD19z T‐cell dose of 1 × 10^9^ – 1 × 10^10^ and an IL2 dose of 100,000 units/kg in two cohorts (Table 1). The median duration of follow‐up was 16.5 months (range 3–53 months). Six out of eight patients (75%) achieved objective responses (all were partial responses), with the duration of response ranging from 3–28 months (Figure 1b). The median progression‐free and overall survivals were 5.3 and 16.25 months, respectively (Figure 1c). Five patients were fit to receive further systemic treatment post progression on CAR T therapy.

**TABLE 1:** Baseline characteristics of patients and their response status.

Patient number	aCD19z dose	Age (gender)	Diagnosis	No prior therapies	Response (duration in months)	Cause of death
002	10^9^	54 (M)	DLBCL	3	PD	Progressive disease
005	10^9^	58 (M)	MCL	8	PR (5.5)	Atypical pneumonia*
006	10^9^	57 (M)	MCL	4	PR (16)	Swine flu
012	10^9^	74 (M)	High‐grade transformation of CLL	3	SD (4.5)	Sepsis*
013	10^10^	62 (F)	DLBCL	1	PR (2)	lymphomatous meningitis
015	10^10^	61 (M)	MZL	3	PR (28)	Progressive disease
016	6 × 10^9^	53 (M)	High‐grade transformation of FL	2	PR (3)	Progressive disease
017	10^10^	51 (M)	High‐grade transformation of FL	7	PR (6)	Progressive disease

* Patient 005 received Lenalidomide post‐CAR T therapy with partial response, patient 012 received Ibrutinib post‐CAR T therapy.

Abbreviations: CLL, chronic lymphocytic leukemia, DLBCL, diffuse large B cell lymphoma; FL, follicular lymphoma, MCL, mantle cell lymphoma; MZL, marginal zone lymphoma.

Quantitative polymerase chain reaction (qPCR) analysis of peripheral blood samples detected aCD19z T cells in both cohorts. Levels peaked at days 5–7 post aCD19z T cell infusion (cohort 1 peak 17.5% of total cells, cohort 2 peak 45% before falling to lower levels). All patients revealed persisting low‐frequency levels at week 6; 3 patients at up to 30 weeks (Figure 1D,E). Two patients received a further course of low‐dose IL2 at week 6, resulting in a transient increase in aCD19z T cell levels. The percent of transduced cells peak was not significantly correlated with the percent of tumour reduction (Spearman's *r* = 0.05, *p* = 0.91). We used fluorescently tagged antibodies to look for dual expression of the T cell CD3 complex and tCD34, which is being used as a marker for the transduced cells. The levels identified were significantly correlated to the qPCR (Spearman's *r* = 0.8, *p* = 0.02). One patient developed central nervous system (CNS) progression and succumbed to his disease. On post‐mortem analysis, the extracranial disease almost disappeared apart from a small fragment of residual lymphoma in one lymph node and in both kidneys. Interestingly, we performed qPCR on the tumour tissues and other healthy tissues. We detected the aCD19z T cells exclusively in tumour tissue and peripheral blood (Supplementary Information File). These data suggest either proper trafficking and selective homing of the aCD19z to the tumour tissues or a potential survival advantage of the aCD19z cells within the tumour tissue.

The treatment was well‐tolerated; two patients developed CRS grade 2, and one developed ICANS grade 1 that resolved in less than 48 h with supportive measures. Otherwise, all patients experienced anticipated transient grade 1–4 hematological toxicities attributable to pre‐conditioning chemotherapy and IL2 that were resolved and managed conservatively in ward‐based settings (Supplementary Table S2).

Taken together, this is the first report on a CAR T cell therapy with a single activating domain that showed meaningful clinical efficacy with two patients achieving durable responses up to 28 months with a favourable safety profile. Interestingly, the magnitude of adverse events observed was less than that generally documented in clinical trials involving second‐generation CAR T cells [6, 7]. Putatively, this may imply the costimulatory domain of the latter generation CAR T as contributing to the toxicity of the T cell product. This is a proof‐of‐concept of the durable clinical efficacy of single activating domain CAR T warranting further investigation, especially for patients who developed severe toxicities on the second and third‐generation anti‐CD19 CAR T cells.

## AUTHOR CONTRIBUTIONS

FT and RH designed the trial. KSS, MP, SC and FT conducted patient recruitment, provided patient care in the trial and contributed to the collection of patient clinical data. RG, DR, DB, NK, DG and RH took oversight of cellular product manufacturing. RG, DR, DB, KSS and MP compiled and analysed all data. KSS performed statistical analyses and wrote the manuscript. All authors have reviewed and approved the manuscript.

## CONFLICT OF INTEREST STATEMENT

FT: Scientific Advisory Board member (T‐Knife), Advisory Board Honoraria (GSK, Ixaka, Janssen, Lucid Bio, Pfizer, Scenic Biotech). RH: Founder and Head of R&D (Instil Bio). DG: stock options (Celyad Oncology) and share holder (Instil Bio). The other authors have no competing interests.

## FUNDING INFORMATION

The Christie NHS Foundation Trust and Cellular Therapeutics Limited

## ETHICS STATEMENT

The study received Research Ethics Committee (REC) favourable opinion and Health Research Authority (HRA) approval (REC ref GTAC103). All patients enrolled provided written informed consent.

## Supporting information

Supporting Information

## Data Availability

Anonymized individual patient data are provided in Table 1 and the Supplementary Information file.
