# Supplementary material for: Evidence of clinical efficacy of a first generation CD19 CAR T cell in B cell malignancies
Source: EJHaem. 2023 Jun 24;4(3):882–5. doi: 10.1002/jha2.731 (PMC10435680; doi:10.1002/jha2.731)
Supplement: Supplementary file 1 — Supporting Information [file JHA2-4-882-s001.docx]

**Supplementary Information File**

**Evidence of clinical efficacy of a first generation CD19 CAR T cell in B cell malignancies.**

**Shohdy KS, et al.**

**Supplementary Notes**

**Pre-clinical evidence**

We have previously published evidence of in vitro activity of our in-house engineered anti-CD19 CAR T cells (Cheadle et al. 2005). Briefly, we have tested T cells engineered to express anti-CD19 scFv CARs. The scFv was isolated from the murine hybridoma HD37 (Pezzutto et al., 1987) by PCR of the heavy and light chains using standard methods (Chester et al., 1994). The scFv was used to generate a fusion with the CD3ζ receptor (Cheadle et al 2005, Gilham et al 2002) and inserted into the rkat vector upstream of an IRES element, which enabled the co-expression of the eGFP marker gene to permit ease of identification of the transduced T cells. Human T cells were found to express intact CAR protein of the predicted molecular mass as demonstrated by immuno-blotting (Cheadle, et al 2005). T cells isolated from 9 patients with a variety of subtypes of NHL were successfully engineered to express the CAR and expanded in vitro (Cheadle et al 2005, Gilham et al 2002). T cells from all 9 patients demonstrated specific cytokine release and cytotoxic activities against CD19+ Raji Burkitt lymphoma cell lines. In addition, T cells from 7 of the 9 patients successfully killed autologous tumour cells in cytotoxicity assays. The biopsies of the remaining two patients whose T cells did not kill the tumours were found to be only 10% and 0% CD19+. In fact there was a clear correlation between levels of CD19 expression on the tumour and functional killing by the engineered T cells.

In vivo activity of human T cells is difficult to assess fully in animal models because these cells are likely to home less effectively due to the inability of human cells to optimally utilise murine adhesion and other key molecules. However, a statistically significant increase in survival (P=0.0016, log rank analysis) has been noted in SCID-Beige mice injected with engineered T cells mixed with Raji cells compared to animals treated with mock engineered T cells mixed with Raji cells.

**Vector design**

The vector we used in the in vitro and in vivo studies contains GFP under the control of an IRES element downstream of the chimeric receptor. Unfortunately, because GFP would render the cells immunogenic in vivo it is not suitable for use in clinical trials. It is, however, crucial to be able to determine expression of the CAR, both for the production of the retroviral producer clones and for analysis of the efficiency of T cell transduction. We have therefore examined ways to achieve this without the need for GFP. Unfortunately, it has not been possible to use CD19 protein directly as a CD19hFc fusion protein produced within the laboratory can not be purified to high enough concentrations to be detected by flow cytometry in conjunction with a PE labelled anti-hFc secondary antibody. We have therefore chosen to make use of a system using truncated CD34 (tCD34) as a marker for the selection of the gene-modified cells.

The tCD34 molecule is a naturally occurring splice variant of CD34 that lacks most of the intracellular signal transduction domain, including the recognition sites for protein kinase C. The residual cytoplasmic tail is thought to play an important role in anchoring the transmembrane protein at the cell surface and has not been reported to be involved in signal transduction (Fehse, et al 2000). Retroviral expression of tCD34 in murine haematopoietic stem cells has not been found to produce overt alterations in multilineage haematopoiesis in the observed 97 weeks (Fehse et al 2002, Li et al. 2002). In addition, T cells expressing tCD34 were found to have normal tissue distribution. The human origin of tCD34 means that it is unlikely to be immunogenic, and importantly, facilities exist for the GMP grade clinical enrichment of cells expressing tCD34 to enable populations of cells expressing tCD34 and hence the CAR to be isolated if required. This is of particular interest in this protocol for the purification of low-level persisting engineered T cells in patients post-therapy for in vitro functional assays.

We have chosen to co-express tCD34 as a marker with the CD19 CAR by using the 2A cleavage system which, unlike IRES, allows the two gene products to be translated in equal efficiency (Klump et al 2001). In addition, we used the pMP71 retroviral vector in the clinical trial rather than rkat since pMP71 is already developed for clinical use (GTAC 96: Phase I Study of MFEz T cells in CEA Positive Tumours). The tCD34.2A.CD19 construct was codon optimisied for human codon usage and synthesised by Genscript Corp and cloned into the pMP71 vector.

We have shown that T cells can regularly be transduced to level of around 40% with the pmp71.tCD34.2A.CD19CD3ζ vector (**Supplementary** **Fig. 1**). Western blot analysis has demonstrated that the chimeric receptor is expressed at the expected size of 46kDa when probed with an anti CD3ζ antibody (**Supplementary** **Fig. 2**). Additionally, T cells transduced with this receptor demonstrate cytotoxicity against CD19 expressing targets and secrete IFNγ (**Supplementary** **Fig. 3**). Importantly, transduced T cells, when cultured on anti CD34 antibody, did not produce IFN or IL-2, nor upregulate expression when cultured on anti-CD34 and anti-CD3, confirming the lack of signalling through the truncated CD34 molecule (**Supplementary** **Fig. 4**).

**Supply of aCD19z T cells**

aCD19z T cells was supplied by the University of Manchester Cellular Therapeutics Unit to GMP standards under the Directive of the European Parliament and of the Council that relates to biological medicinal products 2001/83/EC specifically as advanced therapy medicinal product (ATMP) in the amending regulation 1394/2007. A complete certificate of analysis was provided with each batch of aCD19z T cells and was retained in the Investigator Trial File (ITF)/ Pharmacy File.

**Primary assays**

Primary assays aimed to quantify the relative abundance of aCD19z T cells surviving within the circulation. Assays were carried out in line with Good Laboratory Practice (GLP).

*Real-Time PCR:*

In this setting, Real-Time PCR was used to determine the presence of the transgene within the T cell population (i.e. the retroviral genome itself) and provided an estimation of the number of retroviral genomes present (i.e. the copy number). aCD19z specific Real-Time PCR can detect and accurately quantify aCD19z T cells present within a total white cell population down to a frequency of 0.1%.

*Flow cytometry (CD3+CD34+ cell frequency):*

This is a method that used fluorescently tagged antibodies to look for dual expression of the T cell CD3 complex and CD34 which is being used as a marker for the transduced cells. Incubation of these antibodies with a blood sample results in the T cells being specifically bound by the tagged antibodies and these can then be detected using a flow cytometer machine to accurately quantify the relative frequency of T cells present within the total cell population. In addition, this method should allow quantification of the relative frequency of aCD19z T cells, provided CD34 is expressed at a high enough frequency in vivo for detection.

**Post-mortem analysis**

Patient 013 developed neurologic symptoms 5 weeks post T cell infusion and was admitted. Brain imaging was unremarkable. However, CSF analysis showed infiltration with lymphoma cells confirming the diagnosis of lymphatomous meningitis. He received high-dose IV steroids and Intrathecal steroids, and chemotherapy. Although there was a reduction in lymphoma cells in CSF, no clinical improvement was achieved, and the patient died 8 weeks post-T-cell infusion. Radiologic evaluation of extracranial disease showed a significant reduction in tumour burden. We conducted a post-mortem analysis and collected a small fragments of residual lymphoma in 1 lymph node and in both kidneys. We performed qPCR to the collected tumour tissues and other healthy tissues. We detected the aCD19z T cells exclusively in tumour tissue and peripheral blood, and there was no aCD19z T cells in normal tissues (**Supplementary Fig. 5**). These data suggest proper trafficking and selective homing of the aCD19z to the lymphoma tumour tissues.

**Supplementary References**

Cheadle, E.J., Gilham, D.E., Thistlethwaite, F.C., Radford, J.A. & Hawkins, R.E. (2005) Killing of non-Hodgkin lymphoma cells by autologous CD19 engineered T cells. Br J Haematol, 129, 322-332.

Fehse, B., Kustikova, O.S., Li, Z., Wahlers, A., Bohn, W., Beyer, W.R., Chalmers, D., Tiberghien, P., Kuhlcke, K., Zander, A.R. & Baum, C. (2002) A novel 'sort-suicide' fusion gene vector for T cell manipulation. Gene Ther, 9, 1633-1638.

Fehse, B., Richters, A., Putimtseva-Scharf, K., Klump, H., Li, Z., Ostertag, W., Zander, A.R. & Baum, C. (2000) CD34 splice variant: an attractive marker for selection of gene-modified cells. Mol Ther, 1, 448-456.

Klump, H., Schiedlmeier, B., Vogt, B., Ryan, M., Ostertag, W. & Baum, C. (2001) Retroviral vector-mediated expression of HoxB4 in hematopoietic cells using a novel coexpression strategy. Gene Ther, 8, 811-817.

Gilham, D.E., O'Neil, A., Hughes, C., Guest, R.D., Kirillova, N., Lehane, M. & Hawkins, R.E. (2002) Primary polyclonal human T lymphocytes targeted to carcino-embryonic antigens and neural cell adhesion molecule tumor antigens by CD3zeta-based chimeric immune receptors. J Immunother, 25, 139-151.

Li, Z., Fehse, B., Schiedlmeier, B., Dullmann, J., Frank, O., Zander, A.R., Ostertag, W. & Baum, C. (2002) Persisting multilineage transgene expression in the clonal progeny of a hematopoietic stem cell. Leukemia, 16, 1655-1663.

**Supplementary Tables**

**Supplementary Table 1:** aCD19z T-cell release criteria. These assays were performed prior to infusion of the final aCD19z T-cell product.

| **Assessment** | **Day tested** | **Release Criteria** |
| --- | --- | --- |
| T-cell count  (CD3+ve, Annexin-V-ve 7AAD-ve) | 0 | As per cohort dose or as many as are available up to the maximum T-cell dose |
| T-cell viability  (CD3+ve, Annexin-V-ve 7AAD-ve) | 0 | > 50% of the total CD3^+ve^ population |
| Detectable tCD34 expression | -7 | > 20% of the CD3+ve cells  must express *tCD34 |
| CD25 expression | -7 | Transduced cells (CD19z+) should upregulate CD25 expression on Raji cells 2 fold more than non transduced cells (CD19z-) (CD25+ and 7- AAD-ve cells) |
| Sterility – BacT/Alert | -14 & 10 | Negative |
| Mycoplasma (PCR) | -10 | Negative |

**Supplementary Table 2:** Maximum recorded CTCAE grade for adverse effects for treated participants.

| **Adverse effects** | **002** | **005** | **006** | **012** | **013** | **015** | **016** | **017** |
| --- | --- | --- | --- | --- | --- | --- | --- | --- |
| Cytokine Release Syndrome |  |  |  |  |  | 2 |  | 2 |
| Immune effector cell-associated neurotoxicity syndrome (ICANS) |  |  |  |  |  |  | 1 |  |
| Anaemia |  |  | 1 | 3 | 4 | 2 | 3 | 2 |
| Lymphocyte count decreased | 4 | 4 | 4 | 4 | 4 | 4 | 4 | 4 |
| Neutrophil count decreased |  |  |  | 3 | 4 | 2 | 1 | 4 |
| Platelet count decreased | 4 |  |  |  | 4 |  |  | 2 |
| Alopecia | 1 |  |  |  |  | 1 |  | 2 |
| Nausea |  |  | 1 | 2 |  | 1 |  |  |
| Vomiting | 1 |  |  |  | 2 | 1 |  |  |
| Oral mucositis |  |  |  |  |  |  | 1 | 1 |
| Fatigue |  |  |  | 2 |  | 2 |  |  |
| Microscopic haematuria |  |  |  |  |  |  |  | 1 |
| Weight loss |  |  |  | 1 |  |  |  |  |
| Dry mouth |  |  |  | 1 |  |  |  |  |
| Diarrhoea |  |  |  | 1 |  |  | 1 |  |

**Supplementary Figures**


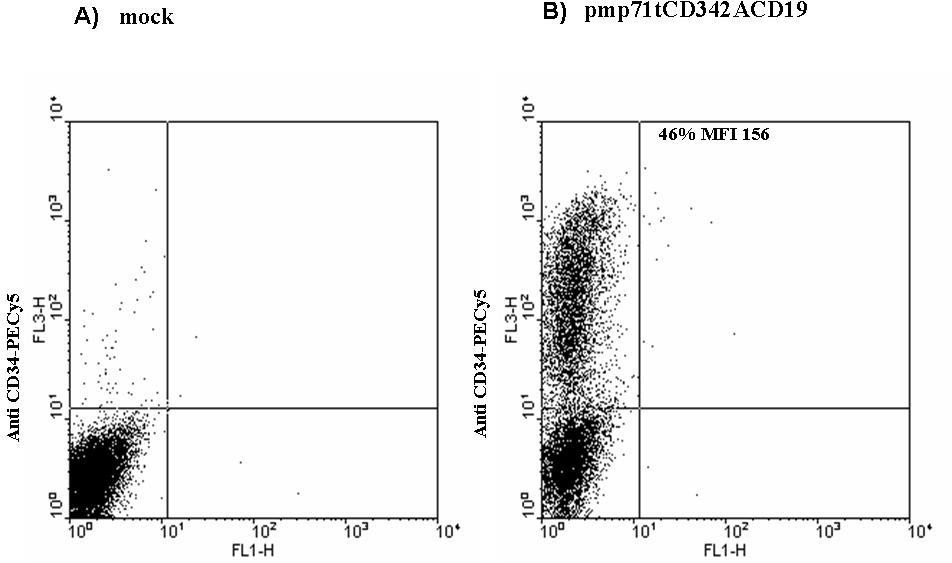


**Supplementary Figure 1:** 1.7x10^6^ T cells were transduced with pmp71.tCD34.2A.CD19 on two consecutive days by spinfection and analysed for expression of the tCD34 by flow cytometry 6 days later. 10^5^ mock (A) or tCD34.2A.CD19 transduced cells were incubated with anti CD34-PeCy5 antibody for 30 minutes at 4 C, washed and 10000 events acquired on a FACscan. Acquired data was analysed using WinMIDI software for CD34 expression.


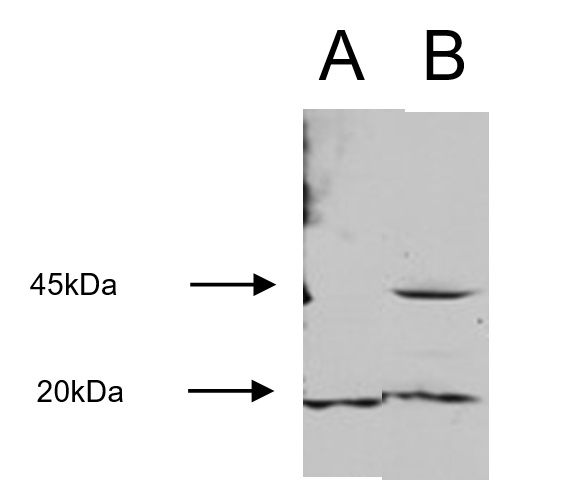


**Supplementary Figure 2**: RIPA lysates of 106 transduced T cells were ran on a 10% polyacrylamide SDS-PAGE gel, transferred to nitrocellulose membrane and probed with mouse anti human CD3 . Following incubation with sheep anti mouse IgG HRP CD3 protein was detected by chemiluminescence. A=mock PBL. B= pmp71tCD34.2A.CD19opt (50%).


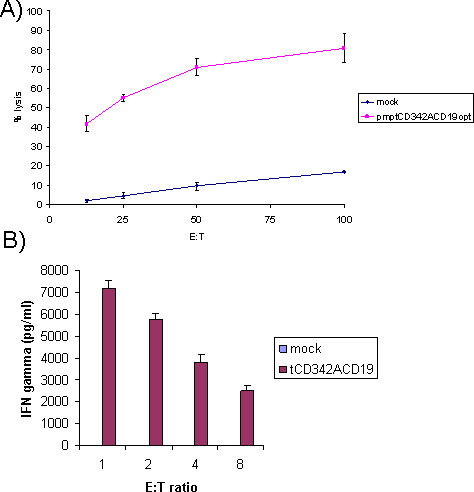


**Supplementary Figure 3:** Functional activity of the codon optimised chimeric receptor. pmptCD342ACD19opt transduced or mock T cells were cultured with 5000 51Cr labelled CD19+ Raji cells at various effector to target (E:T) ratios for 6 hours in U bottomed 96 well plates and cytotoxicity measured by chromium release assay (A). 105 pmptCD342ACD19opt transduced or mock T cells were cultured with decreasing numbers of CD19+ Raji cells for 24 hours in U bottomed 96 well plates and supernatants measured for IFN by solid phase ELISA (B). Note IFNγ levels for mock Transduced cells were unrecordable in all cases.


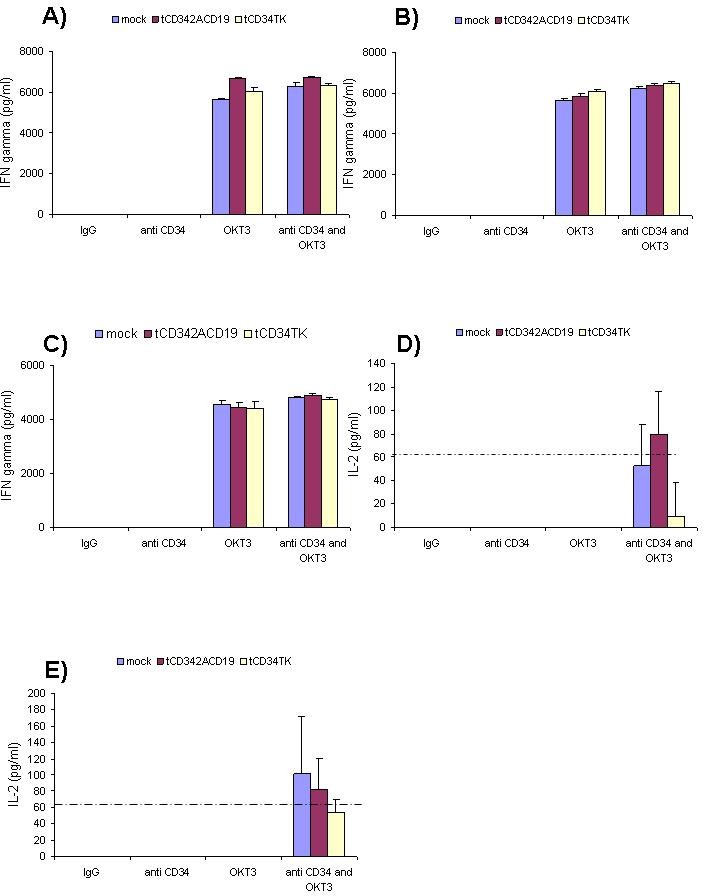


**Supplementary Figure 4**: 106 T cells were cultured on 1 g/ml mouse IgG1 Isotype control, anti human CD34, OKT3 or anti human CD34 and OKT3 monoclonal antibody coated 24 well plates. T cells were either mock transduced or transduced with the pmp71tCD34.2A.CD19 vector or pmp71.tCD34thymidinekinase as a control. After 24 hours (A and D), 48 hours (B and E) and 72 hours (C) supernatants were analysed for the presence of IFN gamma (A, B &C) or IL-2 (D &E) by ELISA. The limit of sensitivity for IFN and IL-2 was 62.5 pg/ml (hashed line). No IL-2 was detected after 72 hours (data not shown).


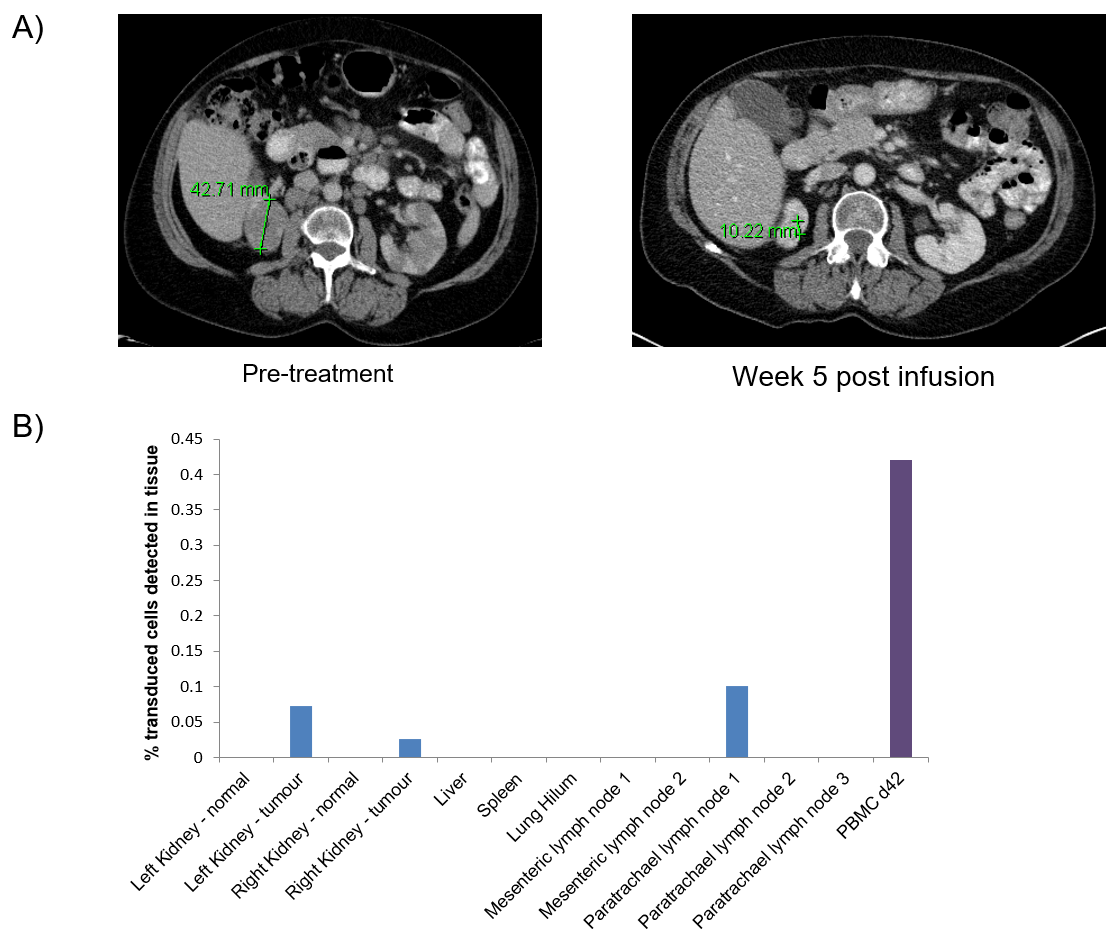


**Supplementary Figure 5:** Radiologic assessment on-treatment and post-mortem analysis of patient 013. A) radiologic scans pre and psot treatment showing major reduction in tumour burden. B) qPCR for transduced cells in post-mortem tissues showing detectable engineered CAR T cells only in the residual lymphoma tissues and peripheral blood and none identified in healthy tissues including (kidneys, liver, spleen, lung).
